# Antagonistic Strain *Bacillus halotolerans* Jk-25 Mediates the Biocontrol of Wheat Common Root Rot Caused by *Bipolaris sorokiniana*

**DOI:** 10.3390/plants12040828

**Published:** 2023-02-13

**Authors:** Kun Kang, Zhipeng Niu, Wei Zhang, Shan Wei, Yangyong Lv, Yuansen Hu

**Affiliations:** College of Biological Engineering, Henan University of Technology, Zhengzhou 450001, China

**Keywords:** *Bipolaris sorokiniana*, *Bacillus halotolerans*, common root rot, biocontrol agent, wheat

## Abstract

Common root rot caused by *Bipolaris sorokiniana* infestation in wheat is one of the main reasons for yield reduction in wheat crops worldwide. The bacterium strain JK-25 used in the current investigation was isolated from wheat rhizosphere soil and was later identified as *Bacillus halotolerans* based on its morphological, physiological, biochemical, and molecular properties. The strain showed significant antagonism to *B. sorokiniana*, *Fusarium oxysporum, Fusarium graminearum,* and *Rhizoctonia zeae.* Inhibition of *B. sorokiniana* mycelial dry weight and spore germination rate by JK-25 fermentation supernatant reached 60% and 88%, respectively. The crude extract of JK-25 was found, by Matrix-assisted laser desorption ionization time-of-flight mass spectrometry (MALDI-TOF-MS), to contain the surfactin that exerted an inhibitory effect on *B. sorokiniana*. The disruption of mycelial cell membranes was observed under laser scanning confocal microscope (LSCM) after treatment of *B. sorokiniana* mycelium with the crude extract. The antioxidant enzyme activity of *B. sorokiniana* was significantly reduced and the oxidation product malondialdehyde (MDA) content increased after treatment with the crude extract. The incidence of root rot was significantly reduced in pot experiments with the addition of JK-25 culture fermentation supernatant, which had a significant biological control effect of 72.06%. Its ability to produce siderophores may help to promote wheat growth and the production of proteases and pectinases may also be part of the strain’s role in suppressing pathogens. These results demonstrate the excellent antagonistic effect of JK-25 against *B. sorokiniana* and suggest that this strain has great potential as a resource for biological control of wheat root rot strains.

## 1. Introduction

Wheat is one of the world’s top three crops, with the second-highest yield after maize [1]. Pathogens associated with common root rots have become one of the major causes of yield reduction and quality loss in wheat, especially durum wheat [2,3]. Common root rot symptoms are characterized by necrotic lesions on the shoot root, usually followed by the shoot crown, that are dark brown to black in color [4]. Root rot infestation is turning into a serious threat to wheat cultivation worldwide, and common root decay has been documented in many wheat-growing locations such as Australia, China, etc. [5,6].

*Bipolaris sorokiniana,* as a typical wheat root rot causal agent, has been reported to be more threatening to durum wheat [4]. *B. sorokiniana* can infest not only the roots of wheat but also the leaves and stems, as well as the wheat grains [7]. In general, there are two aspects of *B. sorokiniana* infestation, one is to reduce seedling establishment by infesting wheat seeds with mold and rot, and the other is to damage various plant tissues, including wheat rootstocks, for the duration of the growth of wheat seedlings [8].

Currently, the most effective way to manipulate root rot in wheat brought on by *B. sorokiniana* infestation is to use chemical control fungicides in combination with agronomic measures [9]. It has been reported that the incidence of root rot in wheat can be significantly reduced by crop rotation of wheat with *Brassica carinata* [10]. Organic agriculture considerably decreases the population of pathogenic fungi linked to root deterioration in wheat soils within a Canadian locale [11]. However, these agricultural practices are costly and require a high degree of mechanized agricultural development, which is not suitable for the actual farming environment and arable land requirements in China. Chemical fungicides are significantly effective in controlling root rot, but they are not in line with the concept of sustainable agriculture because they cause serious pathogenic bacteria resistance and crop chemical residues [12].

In recent years, organic farming techniques have received increasing consideration and the development of effective biocontrol agents is important to complement agricultural management practices and reduce the use of chemical fungicides [4,13]. A number of antagonistic strains inhibiting *B. sorokiniana* have been reported. Several antagonistic strains (e.g., *Trichoderma harzianum*, *Bacillus subtilis*, *Ochrobactrum pseudogrignonense*, *Pseudomonas mediterranea*, etc.) inhibiting *B. sorokiniana* have been reported and these strains obtained by screening from soil are considered safe for the growing environment and mammals [14,15,16,17,18]. Among them, *Bacillus* are considered as the greatest potential to be developed as a biopesticide because of their broad-spectrum antibacterial properties and significant growth-promoting effect. *Bacillus*, as a bacterium, has an advantage over other antagonistic microorganisms in that it has a unique ability to form endospores in response to various adverse environments, and it can proliferate rapidly [19]. Some *bacillus* promote crop growth by producing siderophore and indoleacetic acid as well as playing a remarkable role in the activation of crop immune mechanisms by triggering induced systemic resistance (ISR) [20,21]. It has additionally been reported that *Bacillus* can inhibit the growth of plant pathogenic fungi by producing some extracellular active molecules such as lytic enzymes, antibiotics, and lipopeptides [22]. In conclusion, *Bacillus sp.* as a potential development resource for biocontrol agents has been widely researched. For example, the fermentation supernatant of strain XZ34-1 was reported to be effective in controlling root rot in wheat under greenhouse conditions, as well as having a significant growth-promoting effect [23]. Furthermore, *Bacillus velezensis* LHSB1 inhibited root rot pathogens in peanut through the production of three antimicrobial lipopeptide substances [24].

In this study, we obtained a strain of *Bacillus* sp. JK-25 with a strong antagonistic effect against *B. sorokiniana*. The inhibition effect of this strain against *B. sorokiniana* and the biological control effect in wheat potted plants under greenhouse conditions were investigated. Our research revealed the mechanism of inhibition of *B. sorokiniana* by the antagonistic strain JK-25 and its growth-promoting characteristics.

## 2. Results

### 2.1. Screening of Antagonistic Bacteria

A total of 74 bacterial strains were screened from the collected soil of a wheat field, and five of them showed significant inhibitory effects against common wheat pathogens. Table 1 shows that out of the five bacterial strains, JK-25 exhibited the best antagonistic effect against *B. sorokiniana,* with an inhibition of 82.63%. JK-25 also showed the best inhibition against common wheat bacterial pathogens other than *B. sorokiniana,* including *Fusarium oxysporum, Fusarium graminearum,* and *Rhizoctonia zeae* with inhibition rates of 80.47%, 75.33%, and 59.73%, respectively. Because of the superior inhibition effect of JK-25, with this bacterium having great potential for the biological control of wheat root rot, JK-25 was chosen for further identification and analysis.

### 2.2. Identification of Antagonistic Strain JK-25

**Figure 1:**
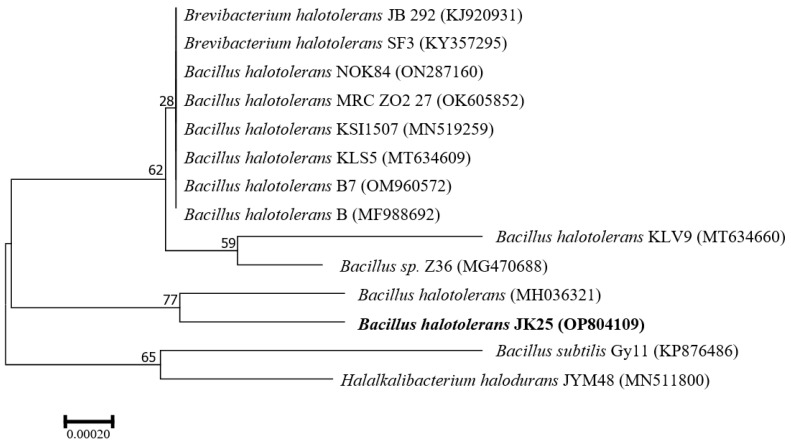
Phylogenetic tree constructed based on 16S rRNA sequences of JK-25. The comparison results were analyzed with MEGA 7.0 software and a tree was built using the neighbor joining method (1000 bootstrap values). Bold is the branch of strain JK-25, whose NCBI registration number is OP804109.

Twenty-four hours after incubation on solid LB medium, strain JK-25 formed round, creamy white colonies without obvious pigmentation, with an overall smooth surface, rough margin and opaque appearance. The strain JK-25 was found to be gram-positive under the microscope and the rod cells contained endospores. It was found to be capable of utilizing citrate and fermenting glucose, reducing nitrate, producing contactase and catalase, hydrolyzing starch and gelatin, and can withstand a high salt environment of 10% NaCl (Table 2). The 16SrDNA sequence of strain JK-25 was uploaded to Genbank (accession number OP804109) and a neighbor-joining phylogenetic tree was constructed (Figure 1). The strain JK-25 showed the highest homology (99.72%) with *B. halotolerans* (accession number MH036321), and the strain was determined to be associated with the cluster of *B. halotolerans*. The morphological analysis, physiological and biochemical tests, and molecular identification of the strain JK-25 were combined to determine that the strain is a *B. halotolerans*.

### 2.3. Inhibitory Effect of the Antagonistic Strain JK-25 in Cultured fermentation broth (CF) on B. sorokiniana 

**Figure 2:**
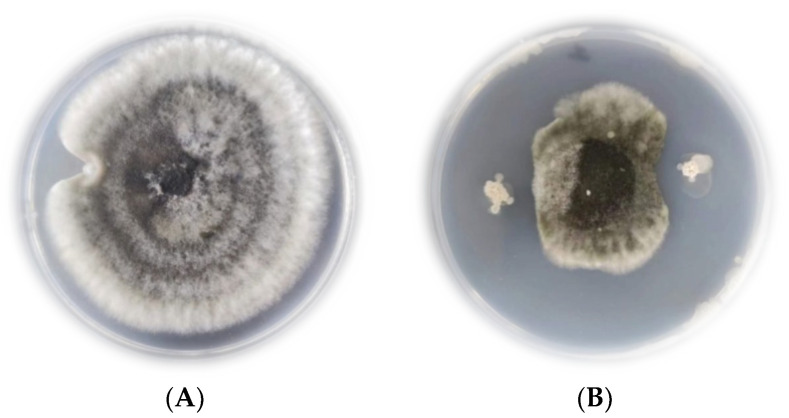
Antagonistic effect of strain JK-25 on *B. sorokiniana* on PDA medium. A: Natural growth of *B. sorokiniana* strain colonies. B: Growth condition of JK-25 spotted around *B. sorokiniana*. Compared with the control (**A**), the dotted antagonist JK-25 could significantly inhibit the growth of *B. sorokiniana* (**B**).

**Figure 3:**
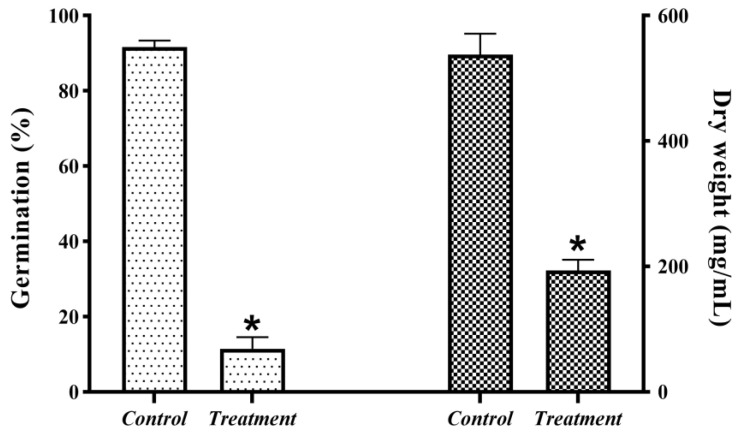
JK-25 exerts an antagonistic effect on mycelial growth and spore germination of *B. sorokiniana.* Data are presented as the average ± the standard deviation and * indicates a significant difference between treatment groups within a parameter (Duncan’s multiple range test at *p* ≤ 0.05).

JK-25 showed a very significant antagonistic effect on *B. sorokiniana* by plate confrontation (Figure 2). The CF of strain JK-25 was found to have a significant inhibitory effect on mycelial growth and spore germination of *B. sorokiniana* (Figure 3). The results in the figure showed a decrease of 63.97% in mycelial dry weight and 87.52% in spore germination rate of *B. sorokiniana* after the addition of JK-25 CF. This indicates that the supernatant contains some antimicrobial substances produced by JK-25 fermentation, which can have a significant antagonistic effect on both mycelium and spores of *B. sorokiniana.*

### 2.4. Detection and Identification of Antifungal Metabolites

**Figure 4:**
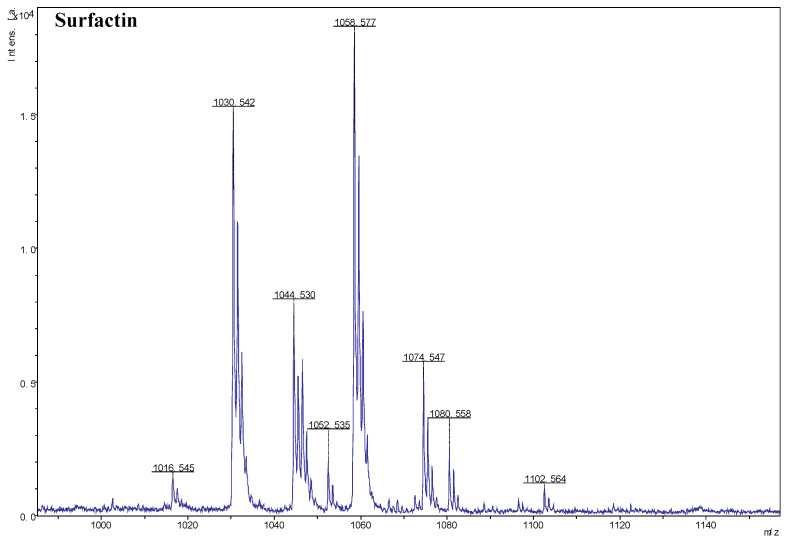
MALDI-TOF mass spectra from *Bacillus halotolerans* JK-25 with the *m/z* range 1000–1140. *m*/*z* represent their corresponding lipopeptides.

MALDI-TOF MS was used for the efficient determination and analysis of the lipopeptides produced by the antagonist strain JK-25. Based on the analysis of the results, surfactin was successfully detected in the crude extract of the fermentation broth. As shown in Figure 4, ions of *m/z* values 1016.545, 1030.542, 1044.530, and 1058.577 were detected, corresponding to C_12–17_ surfactin [M+H]^+^, as previously reported [2], and ions of *m/z* values 1074.547, 1080.558, and 1102.564 correspond to C_14–17_ surfactin [M+Na]^+^. The heterogeneity among peaks differed in molecular weight by 14 Da, suggesting the presence of varied lengths of fatty acid chains within surfactin.

### 2.5. Effect of Crude Extract on Cell Membrane of B. sorokiniana 

**Figure 5:**
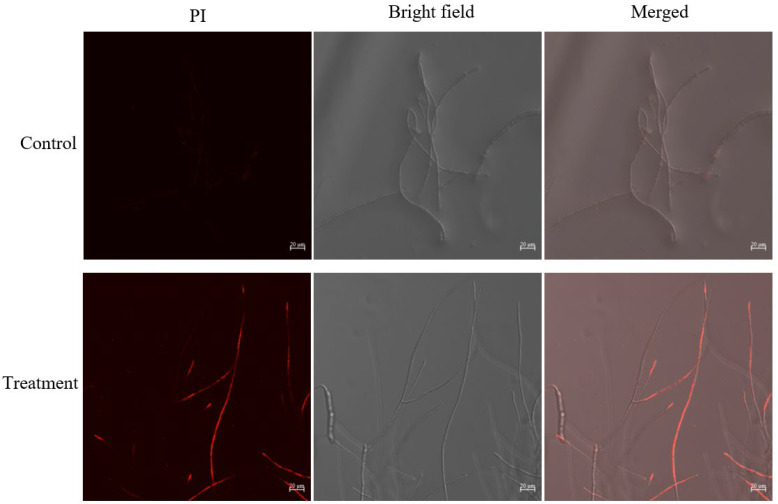
Microscopy analyses of *B. sorokiniana* hyphae treated with JK-25 crude extract. Both control and treated groups were incubated at 30 °C for 12 h and then observed after PI staining.

Under laser scanning confocal microscope (LSCM), it was observed that the surfaces of *B. sorokiniana* mycelium treated with JK-25 crude extract became rougher and many mycelia showed an abnormal twisted state and more branching nodes, while the morphology of untreated *B. sorokiniana* mycelium was more uniform and did not show the above morphology (Figure 5). The treated *B. sorokiniana* mycelium could be observed to have obvious red fluorescence after propidium iodide (PI) staining, while the untreated mycelium did not show fluorescence, indicating that the crude extract of JK-25 significantly disrupted the cell membrane integrity of the mycelium.

### 2.6. Detection of the Effect of Crude Extract on the Antioxidant Activity of B. sorokiniana

**Figure 6:**
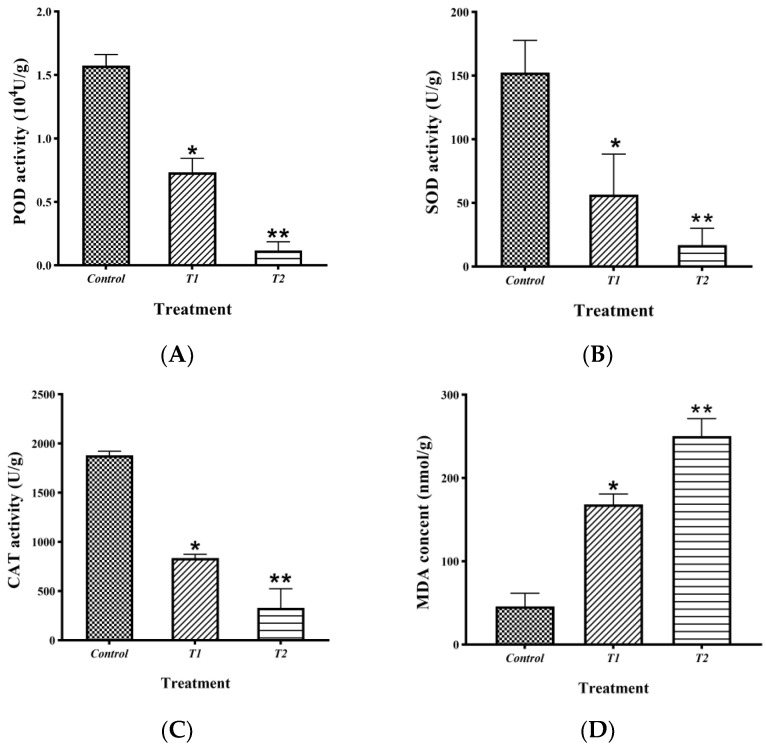
Effect of lipopeptide crude extracts on the antioxidant activity of *B. sorokiniana* mycelia. Control (equal volume PBS treatment), T1 (1% crude extract), T2 (2% crude extract). (**A**) Superoxide dismutase (SOD) activity, (**B**) peroxide (POD) activity, (**C**) catalase (CAT) activity, (**D**) malondialdehyde (MDA) content. Data are presented as the average ± the standard deviation. * and ** indicates a significant difference between treatment groups within a parameter (Duncan’s multiple range test at *p* ≤ 0.05 and *p* ≤ 0.01, respectively).

Activities of superoxide dismutase (SOD), catalase (CAT), peroxidase (POD), and malondialdehyde (MDA) concentrations in the hyphae of *B. sorokiniana* were detected after different treatments with different levels of JK-25 crude extract. Figure 6A–C showed the three aforementioned antioxidant enzyme activities, respectively, and, as can be seen, the enzyme activities in the T1 (1% crude extract) and T2 (2% crude extract) treated groups were significantly lower than those in the control (untreated group). By comparing antioxidant enzyme activities following treatment with different crude extract levels, the reduction in the activities of all three antioxidant enzymes was evident as the crude extract concentration increased. MDA is one of the compounds produced by the oxidative degradation of unsaturated fatty acids on the mycelial membrane system by the action of oxygen radicals. Figure 6D reflects a significant increase in MDA content with increasing crude extract in the treated group compared to the control, indicating that the *B. sorokiniana* mycelial membrane system was disrupted. 

### 2.7. Biocontrol of Wheat Rot under Greenhouse Conditions

**Figure 7:**
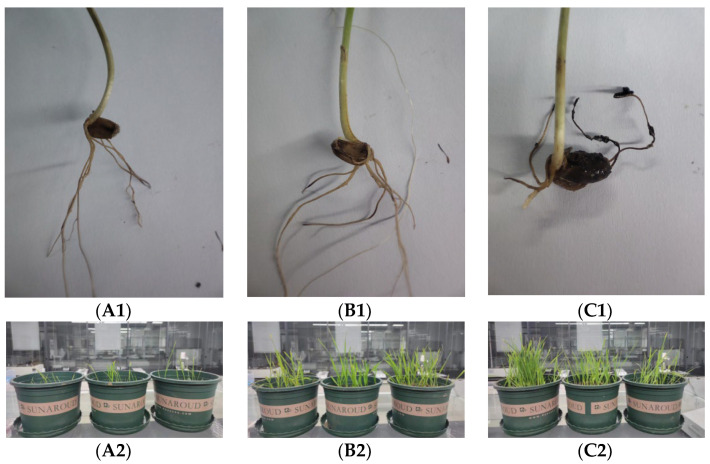
Biocontrol efficiency of JK-25 CF on common root rot caused by *B. sorokiniana*. In all experimental groups, the wheat was pre-inoculated with *B. sorokiniana* during growth. (**A1**,**A2**) Watering with sterile water only during growth; (**B1**,**B2**) irrigation with 50% carbendazim during growth; (**C1**,**C2**) irrigation with JK-25 CF during growth.

Wheat was evaluated for root rot incidence and the effectiveness of biological control after twenty-eight days of growth in pots. As shown in Figure 7(A1,A2), in the control group, the wheat seedlings showed significant wilting and black necrotic lesions (black spots) on the roots after being infected with *B. sorokiniana*. A significant increase in wheat growth status and root health was observed in the treatment groups supplemented with 50% carbendazim solution and supernatant of strain JK-25 fermentation solution (Figure 7(B1,B2,C1,C2)). The addition of JK-25 supernatant significantly suppressed the incidence of root rot compared to the sterile water treatment control, with a biological control effect of 72.06%, although this result was slightly lower than that of the chemical fungicide carbendazim (Table 3). The result suggested that treatment with JK-25 culture broth was useful for the control of wheat common root rot.

### 2.8. Production of Extracellular Enzymes and Growth Promotion

To explore the ability of this strain to produce extracellular enzymes, JK-25 was inoculated onto different types of plates. The results showed that strain JK-25 was able to produce a transparent zone after staining on M9 medium spiked with pectin and cellulose, indicating that the strain was able to secrete pectinase and cellulase. JK-25 was also observed to produce protease and siderophores (Table 4). However, the strain was unable to hydrolyze chitin on the plate, suggesting it could not produce chitinase. The pink color did not appear for indole acetic acid (IAA), indicating that JK-25 does not have the ability to produce IAA.

## 3. Discussion

Common root rot diseases of wheat can lead to significant reductions in wheat yields and cause huge economic losses. It is currently mostly controlled with chemical fungicides but this is not in line with the concept of green agriculture and sustainable development. More and more biological control methods have been applied in the control of wheat root rot in recent times. Sharma et al. reported that actinomycetes isolated from wheat seeds were effective in suppressing soil-borne diseases caused by pathogens including *B. sorokiniana* [25]. Yi et al. reported that *Bacillus amyloliquefaciens* XZ34-1 could inhibit *B. sorokiniana* mycelia dry weight and spore germination through culture filtrate [23]. In the current study, five bacterial strains with an antagonistic effect against *B. sorokiniana* were screened from wheat soil, among which JK-25 showed the highest inhibitory activity against the pathogenic bacterium *B. sorokiniana*. JK-25 also showed an inhibitory effect on *B. sorokiniana* mycelial dry weight and spore germination in this study. 

Different species of antagonistic bacteria inhibit pathogenic bacteria and promote plant growth in different ways, and analysis of the specific mechanisms of action of strain JK-25 could help to improve its biological control in wheat crops. *Bacillus* is a potential antagonist strain due to its ability to suppress plant pathogens by producing a variety of secondary metabolites and antibiotics [26,27,28,29]. Many studies have reported the production of lipopeptides by *Bacillus* as an effective biocontrol agent against plant pathogens. For instance, *Bacillus atrophaeus* FA12 and *B. cabrialesii* FA26 were able to co-produce the lipopeptides iturin and fengycin, which were responsible for the effective antifungal effects against *Xanthomonas oryzae pv. oryzae* [30], while mycosubtilin was shown to be responsible for the biocontrol activity of *Bacillus subtilis* strain Z15 against plant pathogen *Verticillium dahliae* 991 [31]. By analyzing the genome of the strain, Villa-Rodriguez et al. found that *Bacillus cabrialesii* TE3^T^ was able to encode the production of surfactin, fengycin, and rhizocticin A to effectively suppress *B. sorokiniana* [32]. Some reports have also analyzed the ability of an antagonistic strain to produce multiple antimicrobial lipopeptides that act synergistically, enhancing the antimicrobial effect of the strain and broadening the spectrum of inhibition [28,33,34]. Our results show that analysis of the crude extract of JK-25 by MALDI-TOF-MS revealed the presence of surfactin, which is a key substance in the antagonism of *B. sorokiniana* by JK-25. It has also been reported that surfactin can trigger the Induced Systemic Resistance (ISR) in plants to enhance self-protection mechanisms and thus strengthen their own antagonistic effects against phytopathogens [35]. An important mechanism of action of the lipopeptides produced by *Bacillus* sp. to inhibit the growth of plant pathogens is to disrupt their cell membrane system [36]. In this study, significant red fluorescence was observed in the cell membranes of *B. sorokiniana* mycelia treated with crude extracts after PI staining and cell membrane integrity and function were disrupted, which is consistent with the findings reported previously.

Antagonistic bacteria are able to secrete secondary metabolites that disrupt the cellular integrity of pathogens and destabilize their antioxidant systems [37]. The crude extract of the fermentation broth of *Bacillus velezensis* HY19 significantly reduced the enzymatic activities of CAT, SOD, and POD of the pathogenic fungus, thereby inhibiting its growth [38]. Reactive oxygen species (ROS) can cause oxidative damage to biological cells through peroxidation and antioxidant enzymes can scavenge ROS to maintain cellular stability [3]. Reduced antioxidant enzyme activity after treatment of pathogen mycelium with crude extracts of fermentation broth, leading to the accumulation of ROS, can cause destruction of pathogen cells [39]. Therefore, as oxidative damage intensifies, the cell membrane system is disrupted and the level of malondialdehyde (MDA), one of the main products of the oxidation of lipid substances in the membrane system, increases [40]. In our results, after treatment of *B. sorokiniana* mycelium with different levels of crude extracts of strain JK-25, the mycelial antioxidant enzyme activity was significantly reduced compared to the control and the MDA content was significantly increased, which is consistent with previous studies. The increase in mycelial MDA content after treatment also further explains the disruption of mycelial cell membranes observed under the microscope as a result of oxidative damage. 

In the present study, the potential of JK-25 as a highly effective resource of biocontrol was demonstrated by the 72.06% control effect of common root rot in wheat pot experiments conducted under greenhouse conditions. Siderophores have a high affinity for Fe^3+^ and are capable of binding Fe^3+^ to form complexes, which helps Fe uptake by microorganisms, as well as being taken up by plants to increase iron content within the tissues and promote plant growth [41]. A study by Mageshwaran et al. revealed that one of the factors that *Bacillus subtilis* TRO4 promotes in the growth of chickpea plants is the production of siderophores [42]. In line with previous reports, the ability of JK-25 to produce siderophores was verified in this study by the CAS blue agar plate method as one of the reasons for the ability to promote wheat growth. The ability of antagonistic bacteria to act on fungal cell structures, generally through the secretion of extracellular hydrolases, is one of their important mechanisms of action. Li et al. reported that *Bacillus velezensis* HY19 is capable of producing proteases, pectinases, and cellulases to act on fungi to achieve disease control [38]. The present investigation showed that strain JK-25 could produce pectinase and protease. These hydrolases possible enhancement might reduce the ability of the pathogen to invade the wheat tissues of the host. Overall, the antagonistic mechanism of action of strain JK-25 against common root rot in wheat is mainly the production of inhibitory secondary metabolites that directly affect the growth and disrupt the cellular stability of *B.sorokiniana*. In addition, the bacterium enhancing the plant’s protection against the pathogen after infection by triggering ISR of wheat should be tested in the future.

## 4. Materials and Methods

### 4.1. Materials

Soil samples for screening the isolated antagonistic bacteria were collected from arable land where wheat is grown year-round in Zhoukou, Henan Province. The wheat pathogens including *B. sorokiniana, Fusarium graminearum, Fusarium oxysporum,* and *Rhizoctonia zeae* involved in this study were all kept in the laboratory of the School of Biological Engineering, Henan University of Technology. The medium was LB medium (0.5% yeast extract, 1% peptone, 1% NaCl, solid medium with 2% agar, pH 7) and potato dextrose agar (PDA) medium (2% glucose, 20% potato, 2% agar, pH 7).

### 4.2. Isolation and Screening of Antagonistic Bacteria

The sample soil was obtained from the target area using a five-point sampling method at a depth of approximately 10 cm from the surface and was then quickly transferred to the laboratory in sterile bags for temporary storage in a −20 °C freezer. Soil samples were prepared in different concentrations of sample suspensions using the serial dilution method, followed by three dilutions of 10^−5^, 10^−6^, and 10^−7^ in LB medium for coating and incubation, and the individual distinct colonies were selected for numbering and preservation [43].

The plate confrontation method was used for screening the strains for an antagonistic effect against wheat pathogenic fungi [44]. Briefly, the wheat root rot-causing fungus was pre-cultured on PDA medium at 30 °C for 5 d, and a 0.5 cm diameter plug taken from pathogens was placed at the center of a 9 cm diameter PDA plate. Then, candidate strains were inoculated on the plate 2.5 cm away from the plug. Plates without any other strains were used as controls and all plates were cultured at 30 °C until the colonies in the control plate had grown all over the plate. The inhibition rate (IR) was used to express the antagonistic activity of the screened strains which was calculated using the following equation [24]:

(1) $$\mathrm{IR}\left(\%\right)=\frac{\left(A-0.5\right)-\left(a-0.5\right)}{\left(A-0.5\right)}\%$$

where A is the diameter of the control fungus, a is the diameter of the treated fungus, and the 0.5 is the diameter of the inoculated plug of tested strains. 

The strain with the best antagonistic effect was numbered as JK-25 and saved in 30% glycerol at −80 °C for further analysis.

### 4.3. Identification of Strain JK-25

Morphological analyses, as well as physiological and biochemical tests, were performed and combined with molecular methods to identify antagonistic bacterial species. Morphological analysis is performed mainly to observe the colony characteristics, including color, size, growth characteristics, microscopic morphology, and other indicators. Physiological and biochemical tests were performed according to Bergey’s Manual of Determinative Bacteriology [45].

For molecular distinguishing proof, genomic DNA was obtained from the strain utilizing a commercial bacterial genomic DNA extraction kit (Tiangen, Beijing, China) and the 16SrRNA gene region was amplified using bacterial universal primers 27F (5′-AGA GTT TGA TCA TGG CTC AG-3′) and 1492R (5′-ACG GTT ACC TTG TTA CGA CTT-3′). Polymerase chain reaction (PCR) amplification volumes were 25 µL (12.5 µL of PCR MIX (2×), 5 µL of bacterial DNA, 1 µL of each primer, and 5.5 µL sterile deionized water) with the following conditions: 94 °C for 5 min, 30 cycles at 94 °C for 30 s, 55 °C for 30 s, and 72 °C for 100 s, followed by a final extension at 72 °C for 10 min. Then, the PCR products were sent to Biotech Co., Ltd. (Shanghai, China) for sequencing. The DNA sequences were compared for homology through the BLAST tool of the NCBI “http://www.Ncbi.nlm.nih.gov (accessed on 30 December 2022)” bacterial database. The phylogenetic tree was inferred using the neighbor joining method in MEGA7. 0. 14 (Mega Limited, Auckland, New Zealand)with bootstrap values calculated based on 1000 replicates [46,47].

### 4.4. Inhibitory Effect of the Antagonistic Strain JK-25 Cultured Fermentation Broth (CF) on B. sorokiniana

The antagonistic strain JK-25 was cultured for 24 h at 37 °C and 180 r/min in a 250 mL flask containing 100 mL of LB medium [48]. The obtained culture broth was centrifuged at 10,000× *g* and 4 °C for 10 min to remove the bacterial cells and then the supernatant was filtered through a sterile 0.22 μm membrane and then the sterile culture filtrate was collected and preserved at 4 °C.

The spore solution of *B. sorokiniana* (1 × 10^6^ cfu/mL) was accessed from a 250 mL conical flask containing 97 mL of potato dextrose liquid medium at 2% inoculum. One milliliter of the above culture filtrate was added and then incubated at 30 °C and 180 r/min for 5 days. The treatment with the addition of sterile water served as the control group. The fermentation broth in the conical flasks of different treatments was placed in 50 mL centrifuge tubes at 4 °C and 8000× *g* for 10 min to collect the *B. sorokiniana* mycelial precipitate. The mycelium was washed three times with sterile water, then dried at 55 °C to a constant weight and weighed. The spore germination rate was calculated after incubation of *B. sorokiniana* in spore solution for 6 h.

### 4.5. Detection and Identification of Antagonistic Metabolites 

To investigate the antimicrobial metabolites produced by the antagonistic bacterium JK-25, a sterile culture filtrate was first obtained according to the treatment method described in Section 4.4. The pH of the culture filtrate was adjusted to 2 with 6 N HCl and left to stand at 4 °C overnight to allow the crude lipopeptides to precipitate. After that, the precipitate was collected by centrifugation at 4 °C and 11,000× *g* for fifteen minutes and the collected precipitate was completely suspended by using an appropriate amount of methanol. Then, the pH of the precipitate was adjusted to 7.0 with NaOH and allowed to stand for 12 h. Finally, the supernatant was collected by centrifugation at 1100× *g* for 10 min. The methanol extract was filtered and evaporated to one-tenth of the original volume under vacuum. The crude extract was obtained by suspending samples in methanol and performing filtration (0.22 µm, Nylon) [49,50]. 

The above crude extract of the fermentation broth was further analyzed for antibacterial metabolites using MALDI-TOF-MS, referring to the previously reported method [51]. Mass spectrometry was performed on a Bruker Daltonik Reflex MALDI-TOF with a 337 nm nitrogen laser for desorption and ionization using α-cyano-4-hydroxycinnamic acid as a matrix [30,46].

### 4.6. Effect of Crude Extract on Cell Membrane of B. sorokiniana 

The effect of the antagonist JK-25 crude extract on *B. sorokiniana* cell membrane integrity was assessed by propidium iodide (PI) staining. The *B. sorokiniana* was inoculated in a 250 mL conical flask containing 100 mL of PDB medium and incubated at 28 °C for 72 h. The culture medium was centrifuged at 8000× *g* for 10 min to collect the mycelial sediment and the appropriate amount of mycelium was incubated at 28 °C for 12 h. Following this, it was resuspended with 900 μL PBS and 100 μL bacterial crude extract. The treatment with an equal volume of LB medium was used as the control group. The treated mycelial precipitates were collected by centrifugation at 8000× *g* for ten minutes and resuspended with 1 mL PBS, then stained with PI dye at 37 °C in the dark for 20 min. After staining, all treated groups were washed twice with PBS and then observed under laser scanning confocal microscopy.

### 4.7. Detection of the Effect of Crude Extract on the Antioxidant Activity of B. sorokiniana 

The effects on the antioxidant activity and the inhibition mechanism of antibacterial substances were investigated by measuring the activities of superoxide dismutase (SOD), peroxidase (POD), catalase (CAT), and malondialdehyde (MDA) in *B. sorokiniana* mycelium treated with different levels of lipopeptide crude extracts. Briefly, 0.2 g of *B. sorokiniana* mature mycelium was taken and added to 1% and 2% lipopeptide crude extract liquid as described before, respectively, and PBS was supplemented to 1 mL and incubated for 2 h at 28 °C. The treatment group with only 1 mL of PBS added was used as a control and the experiment was repeated three times for each treatment group. The mycelial sediment was collected by centrifugation at 4 °C and 10,000× *g* for 10 min and ground well in liquid nitrogen. The SOD and POD enzyme activities, as well as the MDA content, were determined by referring to the previous report by Yi et al. [23]. The CAT enzyme activity was measured by referring to Zhou et al. [52]. 

### 4.8. Pot Experiments 

Pot experiments used wheat seeds of the Zhengmai 103 variety (purchased from Henan Qiule Seed Technology Co., Ltd., Henan, China). The efficacy of the antagonistic bacterium JK-25 for the control of common root rots was evaluated in wheat pot experiments under greenhouse conditions. Before sowing, the wheat was soaked in 0.5% sodium hypochlorite for half an hour to disinfect the surface and washed twice with sterile water, then sown in plastic pots (15 cm in diameter) containing 3.5 L of soil. Potted plants were grown for 14 days after sowing in greenhouse conditions (25–28 °C and 30–70% for temperature and humidity, respectively). After 14 days, a root rot spore solution (1 × 10^6^ cfu/mL) was irrigated near the soil of wheat roots in pots, followed by 20 mL of bacterial culture filtrate. An equal volume of 50% chemical fungicide, diluted 1000 times, was added as a positive control. A treatment with an equal volume of sterile water was added as a negative control. Six parallel trials were conducted for each treatment group, with each potted replicate guaranteed to have 10 or more wheat plants.

Ten days after treatment growth, all wheat roots were uprooted and washed under a tap with running water for assessment of disease severity. There were five score levels: level 0 for healthy and disease free, level 1 for ≤25% disease, level 2 for >25% to 50% disease, level 3 for >50% to 75% disease, and level 4 for >75% disease. The disease incidence rate (DIR), disease index (DI), and control efficacy (CE) were calculated as follows [53]:

(2) $$\mathrm{DIR}\left(\%\right)=\frac{n}{N}\times 100$$

(3) $$\mathrm{DI}=\frac{\sum_{i=1}^{4}\left(N_{i}\times i\right)}{N\times 4}\times 100$$

(4) $$\mathrm{CE}\left(\%\right)=\frac{{\mathrm{DI}}_{\mathrm{ck}}-{\mathrm{DI}}_{t}}{{\mathrm{DI}}_{\mathrm{ck}}}\times 100$$

where n indicates the number of wheat seedlings with infected roots, N is the total number of surveyed wheat seedlings, N_i_ denotes the number of infected wheat seedlings of a certain level of disease, i denotes a certain level of disease, 4 denotes the highest level of disease, DI_ck_ denotes the disease index of the control group, and DI_t_ denotes the disease index of the treatment group.

### 4.9. Production of Extracellular Enzymes and Growth Promotion

Plate test methods for assessing the growth-promoting properties of the antagonistic strain JK25 includes the production of indoleacetic acid (IAA), extracellular enzymes (protease, pectinase, cellulase, and chitinase), and siderophores. The IAA was determined by referring to the method of Alfiky et al. and extracellular enzymes were detected by the methods described by Khedher et al. [46,54]. Colonies of strain JK-25 were inoculated on CAS blue agar to determine siderophore production [55]. 

### 4.10. Statistical Analysis

Each experiment was performed at least three times and the results were expressed as mean ± standard deviation. All data were analyzed using SPSS 22.0 with Duncan’s new multiple range test, where *p*-values < 0.05 were considered statistically significant. All statistical analyses were carried out with the help of Graph pad prism software (version 9.1.2, graphpad Software, San Diego, CA, USA).

## 5. Conclusions

*B. halotolerans* JK-25 showed a very pronounced antagonistic effect against *B. sorokiniana* and broad-spectrum resistance to wheat pathogens. This strain has shown significant biocontrol effects on a plant pathogen in an experiment using wheat in pots under greenhouse conditions and has revealed a preliminary mechanism of action. Further analysis revealed that the strain had phytopromotional properties including the ability to produce substances such as siderophores. The presence of lipopeptides, including surfactin, in the crude extract of JK-25 fermentation broth was detected by MALDI-TOF-MS analysis. The antioxidant capacity of *B. sorokiniana* treated with JK-25 crude extract was significantly reduced and oxidative damage increased. In summary, the antagonistic strain JK-25 has good potential for use in the biological control of common root rot caused by *B. sorokiniana* in wheat and needs further research and development.

## Figures and Tables

**Table 1:** Inhibition rate of pathogens by five strains of bacteria.

Strain	Inhibition Rate (%)			
	*Bipolaris* *sorokiniana*	*Fusarium* *oxysporum*	*Fusarium* *graminearum*	*Rhizoctonia* *zeae*
JK-5	78.38 ± 2.63 ab	76.97 ± 1.22 a	69.63 ± 1.46 b	25.73 ± 7.9 c
JK-13	75.4 ± 2.79 b	71 ± 2.08 b	65.2 ± 2.75 bc	45.7 ± 2.21 b
JK-25	82.63 ± 0.67 a	80.47 ± 1.4 a	75.33 ± 1.27 a	59.73 ± 2.45 a
JK-44	66.06 ± 2.07 c	57.2 ± 1.97 c	62.47 ± 3.2 c	30 ± 8.94 c
JK-58	67.2 ± 3.44 c	55.3 ± 3.22 c	64.87 ± 3.33 bc	32.47 ± 2.54 c

Values in the table are the means ± standard deviation of the results of at least nine replicates. Different letters indicate significant differences after Duncan′s multiple range test (*p* ≤ 0.05).

**Table 2:** Physiological and biochemical characteristics of antagonistic strain JK-25.

Biochemical Tests	Reaction	Colony Morphology	Description
Gram stain	+	Endospores	Present
Methyl red test	−	Morphology	Rounded
Voges-Proskauer test	+	Pigment	Creamy white
Indole test	−	Surface	Neat and smooth
Nitrate reduction	+	Margin	rough
Catalase	+	Opacity	Opaque
10% salt tolerance test	+		
Glucose fermentation	+		
Starch hydrolysis	+		
Citrate test	+		
Gelatin liquefaction	+		

“+” Positive; “−” negative.

**Table 3:** Biocontrol efficacy of JK-25 on common root rot.

Treatments	Disease Incidence Rate (%)	Disease Index	Control Efficacy (%)
Sterile water control	98.75 ± 3.81 a	71.59 ± 7.24 a	-
Carbendazim control	42.37 ± 8.96 c	15.85 ± 2.41 b	77.86 ± 4.32
CF treatment	49.65 ± 9.54 b	20.20 ± 3.58 b	72.06 ± 6.94

Values represent means ± SDs. The presence of different lowercase letters within the same column indicated a significant difference between treatments (*p* < 0.05).

**Table 4:** Identification of JK-25 growth-promoting conditions.

Bioboosters	Reaction
Protease	+
Pectinase	+
Cellulase	+
Chitinase	−
Siderophores	+
Indoleacetic acid (IAA)	−

“+”Positive; “−”negative.

## Data Availability

The relevant data for this study are presented in the article for the reader’s own reference.
